# The Power of Empowerment: Predictors and Benefits of Shared Leadership in Organizations

**DOI:** 10.3389/fpsyg.2020.582894

**Published:** 2020-11-19

**Authors:** Charlotte M. Edelmann, Filip Boen, Katrien Fransen

**Affiliations:** Department of Movement Sciences, KU Leuven, Leuven, Belgium

**Keywords:** shared leadership, empowering leadership, Social Identity Approach, peer leadership quality, team effectiveness, well-being

## Abstract

Leadership plays an essential part in creating competitive advantage and well-being among employees. One way in which formal leaders can deal with the variety of responsibilities that comes with their role is to share their responsibilities with team members (i.e., shared leadership). Although there is abundant literature on how high-quality peer leadership benefits team effectiveness (TE) and well-being, there is only limited evidence about the underpinning mechanisms of these relationships and how the formal leader can support this process. To address this lacuna, we conducted an online survey study with 146 employees from various organizations. The results suggest that an empowering leadership style of the formal leader is associated with higher perceived peer leadership quality (PLQ) on four different leadership roles (i.e., task, motivational, social, and external leader). In addition, formal leaders who empower their team members are also perceived as better leaders themselves. Moreover, the improved PLQ was in turn positively related to TE and work satisfaction, while being negatively related to burnout. In line with the social identity approach, we found that team identification mediated these relationships. Thus, high-quality peer leaders succeeded in creating a shared sense of “us” in the team, and this team identification in turn generated all the positive outcomes. To conclude, by sharing their lead and empowering the peer leaders in their team, formal leaders are key drivers of the team’s effectiveness, while also enhancing team members’ health and well-being.

## Introduction

For many decades, organizational structures were vertically structured with the formal leader being hierarchically placed above the followers. This conceptualization inferred that leadership is a downward process in which a single individual in a team or organization – the formal leader – influences his or her followers (Pearce and Conger, 2003; Bass and Bass, 2008). However, since the beginning of the new millennium, organizations are faced with fast-changing environments and increasing workload with complex tasks (Day et al., 2004). These changes place unrealistic expectations upon formal leaders, as it is unlikely that a single person can effectively perform all leadership responsibilities (Yukl, 2010). As a result, organizations have increasingly started to question this conventional single-leader paradigm.

This debate gave rise to a shared leadership approach, which implies that rather than burdening one individual with all the responsibilities, it is more realistic and effective to rely on the strengths of the team members to share these leadership tasks. The concept of shared leadership has been defined as “an emergent team property that results from the distribution of leadership influence across multiple team members” (Carson et al., 2007, p. 1218). This approach entails that leaders cannot only be formally appointed in their role, with leadership responsibilities being officially and explicitly assigned to them (e.g., managers and directors). Instead, leaders can also emerge as informal leaders due to their natural interactions with their colleagues (Pearce and Conger, 2003).

During the last decade, the interest in shared leadership has substantially increased and the topic receives considerable recognition in performance psychology. Indeed, research in organizational teams revealed a positive impact of shared leadership above and beyond that of vertical leadership structures on a variety of outcomes, including goal commitment, team confidence, and tangible performance indicators such as productivity (e.g., Hoch, 2007; Parker et al., 2015). In particular, the literature focusing on modern shared leadership structures in organizations, such as self-directed and agile teams, points towards the positive impact of shared responsibilities because they foster the sharing of values and norms and generate a stronger sense of team competence (Solansky, 2008; McIntyre and Foti, 2013). Moreover, shared leadership has also been found to buffer against team conflict (e.g., Bergman et al., 2012).

### Role Differentiation

The efficiency of a structure of shared leadership has been argued to hinge upon a transparent definition and allocation of roles (Bray and Brawley, 2002). Bales and Slater (1955), founders of the role differentiation theory, proposed a dual leadership structure including two leadership roles focusing on either task activities (instrumental leader) or socio-emotional activities (expressive leader). A team structure encompassing both an instrumental and an expressive leader was found to minimize time, effort, and psychological tensions between team members (Pearce and Conger, 2003). Throughout time, researchers also suggested considering other leadership roles, such as goal setter, planner, and group symbol as well as coach and promotor of team learning (Krech et al., 1962; Wageman, 2001; Yukl et al., 2002).

Besides these already established suggestions on different leadership roles, a large number of other studies have provided evidence that identifying different roles within an organizational team benefits the team’s performance (Lee et al., 2015). However, it should be noted that most of the studies on role differentiation have focused exclusively on the roles of formal leaders (e.g., Quinn, 1988; Kozlowski and Bell, 2013). Despite numerous calls of scholars in the field emphasizing the need to also identify leadership roles for peer leaders within organizational teams (e.g., Lee et al., 2015), such a set of leadership roles for employees *within* a team is still lacking.

Earlier research findings from the team sports context might provide inspiration to fill this knowledge gap. In this regard, research on peer leadership revealed that athletes in sports teams could occupy more leadership roles than the traditional roles of task and social leadership, outlined by Bales and Slater (1955). First, Loughead et al. (2006) added the role of the external leader, who represents the team towards outer parties, such as club management, media, and sponsors, while also securing desired resources and support as well as buffering team members from outside distractions. Finally, more recent research in the sport context further added the role of motivational leader, who was able to motivate team members to give their very best (Fransen et al., 2014). This resulted in a peer leadership categorization of four leadership roles, including the task, motivational, social, and external leader (for definitions of each of these leadership roles, see Table 1). Noteworthy is that sports teams in which leadership across these four leadership roles was occupied by different team members appeared to perform better than teams relying on one heroic team captain (Fransen et al., 2014). This is in line with the finding that, even though players and coaches expect their team captain to take up these four leadership roles, their captains can only rarely live up to these high expectations (Fransen et al., 2019).

**Table 1:** Means, standard deviations, and correlations between all included (sub)scales and their respective reliability.

S. No.		*M*	*SD*	*α*	1	9	10	11	12	13	14	15	16
1.	Empowering leadership (EL)	5.96	2.25	0.98									
2.	EL – subscale self-reward	4.11	2.52	0.93	0.72^***^	0.34^***^	0.37^***^	0.37^***^	0.41^***^	0.44^***^	0.46^***^	−0.37^***^	0.38^***^
3.	EL – subscale teamwork	6.41	2.36	0.93	0.86^***^	0.54^***^	0.49^***^	0.52^***^	0.53^***^	0.66^***^	0.59^***^	−0.42^***^	0.54^***^
4.	EL – subscale participative goal setting	5.75	2.69	0.96	0.87^***^	0.44^***^	0.38^***^	0.53^***^	0.45^***^	0.58^***^	0.57^***^	−0.42^***^	0.37^***^
5.	EL – subscale independent action	6.63	2.46	0.94	0.89^***^	0.36^***^	0.36^***^	0.33^***^	0.44^***^	0.54^***^	0.53^***^	−0.30^***^	0.41^***^
6.	EL – subscale opportunity thinking	6.02	2.60	0.92	0.93^***^	0.40^***^	0.41^***^	0.49^***^	0.46^***^	0.55^***^	0.50^***^	−0.37^***^	0.41^***^
7.	EL – subscale self-development	6.29	2.64	0.98	0.95^***^	0.45^***^	0.45^***^	0.50^***^	0.54^***^	0.61^***^	0.60^***^	−0.42^***^	0.42^***^
8.	Peer leadership quality (PLQ)	6.72	1.63	0.82	0.63^***^	0.81^***^	0.81^***^	0.80^***^	0.83^***^	0.63^***^	0.58^***^	−0.31^***^	0.52^***^
9.	PLQ – task leadership	6.71	2.07	na	0.48^***^								
10.	PLQ – motivational leadership	6.90	1.93	na	0.47^***^	54^***^							
11.	PLQ – social leadership	6.81	1.88	na	0.52^***^	0.52^***^	50^***^						
12.	PLQ – external leadership	6.60	2.03	na	0.55^***^	0.52^***^	0.57^***^	0.54^***^					
13.	Team identification	5.08	1.25	0.90	0.65^***^	0.58^***^	0.43^***^	0.51^***^	0.54^***^				
14.	Work satisfaction	5.08	1.06	0.87	0.63^***^	0.54^***^	0.41^***^	0.36^***^	0.56^***^	0.69^***^			
15.	Burnout	2.77	1.10	0.90	−0.44^***^	−0.28^**^	−0.27^**^	−0.31^***^	−0.19^*^	−0.42^**^	−0.46^***^		
16.	Team effectiveness (TE)	6.73	1.75	0.94	0.48^***^	0.56^***^	0.41^***^	0.37^***^	0.37^***^	0.69^***^	0.49^**^	−0.24^**^	
17.	TE – subscale output	6.82	1.81	0.91	0.43^***^	0.56^***^	0.35^***^	0.38^***^	0.34^***^	0.64^***^	0.45^***^	−0.24^**^	0.92^***^
18.	TE – subscale quality	6.85	1.92	0.88	0.40^***^	0.50^***^	0.36^***^	0.31^***^	0.27^**^	0.61^***^	0.42^***^	−0.19^*^	0.93^***^
19.	TE – subscale change	6.46	1.98	0.90	0.43^***^	0.54^***^	0.41^***^	0.34^***^	0.33^***^	0.65^***^	0.42^***^	−0.26^**^	0.90^***^
20.	TE – subscale organization and planning	6.69	1.93	0.89	0.44^***^	0.50^***^	0.37^***^	0.32^***^	0.36^***^	0.63^***^	0.47^***^	−0.23^**^	0.93^***^
21.	TE – subscale interpersonal communication	6.01	2.08	0.95	0.43^***^	0.46^***^	0.36^***^	0.34^***^	0.35^***^	0.57^***^	0.39^***^	−0.21^*^	0.85^***^
22.	TE – subscale value	6.81	1.98	0.97	0.41^***^	0.47^***^	0.38^***^	0.30^***^	0.32^***^	0.63^***^	0.40^***^	−0.17^*^	0.86^***^
23.	TE – subscale overall	7.11	1.91	0.96	0.49^***^	0.52^***^	0.41^***^	0.37^***^	0.37^***^	0.68^***^	0.53^***^	−0.24^**^	0.95^***^
24.	Formal leadership quality	5.93	2.08	0.91	0.76^***^	0.57^***^	0.50^***^	0.56^***^	0.55^***^	0.63^***^	0.56^***^	−0.38^***^	0.52^***^

na = Value not available as the scale was restricted to only one item.

* *p* < 0.05;

** *p* < 0.01;

*** *p* < 0.001.

Inspired by the already manifested value of shared leadership in modern organizations, as well as the initial evidence of four critical peer leadership roles in sports teams, this study aims to provide similar insight into peer leadership in organizations. As previous research emphasized that “the principles of elite performance in sport are easily transferable to business contexts” (Jones, 2002, p. 279; Wagstaff, 2017), we will rely on the four-fold categorization of peer leadership in sport settings. The underpinning reason for the similarities between both contexts is that sport and business teams face similar principles of leadership; while both types of teams are usually hierarchically structured with a single formal leader, research in both contexts demonstrated the advantages of leadership being shared among team members. More specifically, to provide a sound basis for further research on the topic, we aim to tackle four research questions in this study.

### Aim 1: How Does Peer Leadership Quality Benefit the Team and Its Members?

While there is broad evidence based on the positive impact of shared leadership on team-level outcomes like TE and confidence (e.g., Pearce and Sims, 2002; Wang et al., 2014; Wu et al., 2020), two lacunae remain. First, most studies measured shared leadership as the degree to which team members occupy leadership responsibilities. In other words, these studies rated people as leaders based on the quantity of leadership behaviors they showed. To obtain this quantification, researchers used methods such as coding videotapes according to predefined leadership behaviors (e.g., Künzle et al., 2010; Bergman et al., 2012) or simulation techniques such as policy-capturing based on hypothetical scenarios (e.g., Drescher and Garbers, 2016). However, this quantitative distinction does not provide us with any information on the quality of their leadership. As Zhu et al. (2018) argued, the current measures of shared leadership only capture its configuration, while the actual content of specific leadership roles, and the performance (i.e., leadership quality) hereof, has been overlooked so far. It should be noted that previous experimental evidence obtained from the sport context showed that peer leaders can also have a detrimental impact on TE (e.g., Fransen et al., 2015a, 2018). In other words, in order to predict the expected benefits of peer leadership, it is essential to take the *quality* of peer leaders into account, rather than the presence or the amount of leadership behaviors.

A second lacuna in the present research on peer leadership is that, while the effects on TE have been extensively studied, the benefits for health and well-being remain unknown. The few studies exploring these outcomes only tackled the health advantages for formal leaders (Lovelace et al., 2007). While research in sport contexts has demonstrated that peer leadership quality (PLQ) also entails benefits for team members’ health and well-being (Fransen et al., 2020a), this relationship has not been established in organizational contexts. Several scholars have acknowledged a potential impact of shared leadership on health outcomes and proposed to further investigate the health and well-being benefits (e.g., Zhu et al., 2018; Sweeney et al., 2019). However, while some studies investigate the relation between shared leadership and health outcomes such as job satisfaction, reduced levels of conflict and job stress (e.g., Shane Wood and Fields, 2007; Wang et al., 2014), the relationships with health at a physical or psychological level have not yet been tested. This is unfortunate as promoting satisfied and healthy employees would be in an organization’s best economic interest (Litchfield et al., 2016).

To address these research lacunae, the present study will investigate the *leadership quality* of peer leaders, more specifically the leadership quality of the best task, motivational, social, and external leader in the team. Furthermore, we will investigate the relationship between PLQ on the one hand and of individual perceptions of both TE and indicators of well-being on the other hand. We expect that the relations found in sports teams will hold for business teams as well.

*H1*: Peer leadership quality on each of the four leadership roles is significantly positively correlated with team effectiveness (H1a) and work satisfaction (H1b), while being significantly negatively correlated with burnout (H1c).

### Aim 2: Is Team Identification the Missing Link?

While most of the research on shared leadership has primarily focused on the investigation of its direct effects, some scholars have also shed light on the mechanisms underpinning this relationship (e.g., Hoch, 2007). Previous research in this regard suggested the potential mediating role of employees’ identification with their team (e.g., Zhu et al., 2017). This suggestion is in line with the social identity approach (SIA, Haslam, 2004), an integrative theoretical framework on (inter)group processes that has been extensively applied to organizations. SIA argues that the behavior of team members is shaped by thinking and behaving in terms of their shared social identity (i.e., as “us, team members”) rather than in terms of their personal identity (i.e., as “you” and “me”). With respect to leadership, the SIA to leadership suggests that leaders are only effective to the extent that they succeed in managing – that is creating, representing, advancing, and embedding – a shared social identity in their teams (i.e., they provide identity leadership; Haslam et al., 2011).

A large body of organizational research has evidenced the resulting benefits of these social identities, including employee performance, team satisfaction, and TE (e.g., Tanghe et al., 2010; Steffens et al., 2014; Reis and Puente-Palacios, 2019). Furthermore, a meta-analysis has shown that when employees identify strongly with their team or organization, this also benefited their health and well-being (Steffens et al., 2017). Several field studies in organizations further demonstrated the impact of perceived identity leadership by the formal leader on lower subsequent burnout among employees (Steffens et al., 2014, 2018). The underlying reasoning is that team identification allows employees to feel supported by their colleagues, thereby contributing to their ability to cope with stress (Haslam et al., 2009). In fact, a systematic review with studies conducted in more diverse applied contexts (e.g., in a community, health/clinical, educational, or organizational setting) revealed that team identification-building interventions benefit a variety of health outcomes, ranging from reduced stress, depression, and anxiety to enhanced well-being as well as cognitive and physical health (Steffens et al., 2020). Similar results have been recently found in the sport setting, where formal leaders as well as peer leaders demonstrating identity leadership, were found to create a psychologically safe environment through which individuals’ burnout is buffered, thereby enhancing their health (Fransen et al., 2020c).

It should be noted, though, that when previous studies incorporated leadership as a predictor in their analysis, this leadership was related to the leadership of the formal leadership (e.g., the manager). To our knowledge, no organizational studies have yet sought to understand the role of team identification in explaining the relationship between informal PLQ and both the TE and member health and well-being. The present study aims to address this gap in the literature. To formulate our hypothesis, we rely again on previous sports research that demonstrated that the importance of identity leadership does not only hold for the coach as a formal leader, but also for peer leaders within the team (e.g., Steffens et al., 2014). More specifically, research has shown that team identification mediated the relationship between high-quality athlete leadership and TE (Fransen et al., 2015a, 2020a). Furthermore, a study with professional football teams revealed that the quality of peer leaders influenced athletes’ health and burnout, but only to the extent that peer leaders were able to increase teammates’ identification with their team (Fransen et al., 2020a). We expect that these relations observed in sport contexts will also hold for organizational contexts.

*H2*: Team identification mediates the relationship between peer leadership quality and team effectiveness (H2a), work satisfaction (H2b), and burnout (H2c).

### Aim 3: The Role of the Formal Leader in Promoting Shared Leadership

Despite the benefits that shared leadership structures can create, little is known about the antecedents that can promote the quality of these peer leaders. Even though research is still in its infancy, the formal leader is thought to play an essential role herein. Extant research suggests that a specific leading style of the formal leader, in particular empowering leadership (EL), facilitates the emergence of shared leadership within a work team (Margolis and Ziegert, 2016; Van Knippenberg, 2017). EL is defined as the extent to which leaders enhance autonomy, control, self-management, and confidence in their team (Chen et al., 2011). In other words, we expect that the more a formal leader engages in behaviors that psychologically empower employees, the more employees will be stimulated to engage in qualitative leadership.

*H3*: Empowering leadership behavior by the formal leader is positively related to higher peer leadership quality within the team.

**Figure 1:**
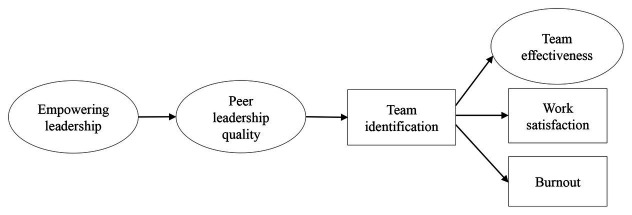
Structural model representing the expected pathways of empowering leadership, peer leadership quality, and team identification as described in H1-4. Empowering leadership, peer leadership quality, and team effectiveness are depicted as latent variables inferred from their subscales, as discussed in the Methods section.

Figure 1 represents the overall model that captures Hypotheses 1, 2, and 3.

### Aim 4: The Barriers Withholding Formal Leaders From Shared Leadership

Despite the benefits that team members can obtain from shared leadership, formal leaders might consider the process of sharing leadership to be a threat to their formal status. According to Zhu et al. (2018), formal leaders can experience “psychological territory infringement” (p. 39). In other words, when team members occupy leadership roles, formal leaders might fear that the development of their own leadership capabilities can be inhibited. Other potential thresholds mentioned in literature are the fear of losing control, being perceived as lazy, or the idea that time-pressuring situations require vertical leadership structures (Ntoumanis and Mallett, 2014). It is important to examine whether these perceived thresholds actually exist or whether they are only fiction. However, as far as we know, no research in organizations has yet investigated the relationship between the quality of peer leadership on different roles and the perceived leadership quality of the formal leader. Preliminary evidence in sports teams suggests that players in teams with high‐ compared to low-quality peer leadership also perceived their coach as a better leader (Fransen et al., 2020d). This finding held for each of the four leadership roles (e.g., the more task leadership quality on the team, the more players perceived their coach to be a good task leader). These findings suggest that when coaches stimulate athletes to engage in leadership responsibilities and thus become better peer leaders, these coaches will also be perceived as better leaders themselves. According to this study, coaches’ fear of losing authority when sharing their leadership cannot be considered justified. We expect that the same conclusion holds for organizational leaders.

*H4*: The leadership quality of the task, motivational, social, and external peer leader is positively related to the perceived quality of the formal leader’s leadership on each of the four roles.

## Methods

### Procedure

The present study was carried out in Belgium and had a cross-sectional, quantitative design. Data were collected by means of an online survey. Participants were required to be at least 18 years old, to be employed in Belgium, and to have a direct supervisor. Therefore, only people working in organizations with hierarchical levels were targeted during data collection, whereas self-employed people without a leader were excluded.

First, human resource managers of organizations, as well as personal contacts (e.g., family, friends, and professional network), were randomly approached and contacted via mail with a written request to participate in a study about leadership and well-being at work. Anonymity and confidentiality were guaranteed and ethical approval for the implementation of this study was obtained from the Social and Societal Ethics Committee at KU Leuven (G-2016 09 630). Participation was voluntary and not reimbursed. However, as a motivational incentive, participation in a lottery was offered with a one-in-five chance of winning a €20 voucher from bol.com, if participants completed the survey and provided their email address. Upon agreement with the human resource manager, the survey was sent to participants’ email address. All items included in this survey were presented in the corresponding language of the participants (i.e., Dutch or French). Both translations of the questionnaires were conducted by native speakers and double-checked by the researchers for grammatical correctness and accuracy of content before distributing the survey.

### Participants

A heterogeneous sample of 146 adult employees working in medium-sized to large organizations located in Flanders and Wallonia participated in this study. More specifically, the organizations mostly belonged to the industries of civil aviation, clothing manufacturing, retail, and education. Participants’ age was retrieved through five age categories that ranged from 18 to 55+ years, with 16.4% of participants being between 18 and 25 years old, 39% of the participants between 25 and 35 years old, 14.4% between 35 and 45 years old, 19.9% between 45 and 55 years old, and 10.3% of the participants being older than 55 years.

In terms of gender, the sample consisted of 54.1% female and 45.9% male employees. Moreover, 76.7% of participants worked full-time, in contrast with the remaining 19.2% of participants working part-time, and 4.1% having another working format such as shiftwork or a mini job. Participants responded that there were on average 14 members in their team (*SD* = 30.8). The general work experience ranged between less than 1 year and more than 20 years with an average of 7 years (*SD* = 1.3). Finally, participants were employed in their present organization for an average of 5 years (*SD* = 1.4).

### Measures

All measures were self-reports. The reliability of all scales and their respective subscales used to test H1, 2, 3, and 4 are reported in Table 1.

#### Empowering Leadership

The 22-item scale by Pearce and Sims (2002) was used with six subscales examining the degree to which the formal leader encourages self-reward, teamwork, participative goal setting, independent action, opportunity thinking, and self-development. These items were rated on an 11-point Likert scale, ranging between 0 (*disagree completely*) and 10 (*agree completely*), with an example item being: “My team leader advises me to coordinate my efforts with other individuals who are part of the team.”

#### Peer Leadership Quality

This variable encompasses the four leadership roles by Fransen et al. (2014), applied to the organizational context (see Table 2). Perceived leadership quality on each of these roles was assessed by presenting the role definition, followed by the instruction “Think of a team member that corresponds best with this role and rate the quality to which he/she fulfills this role.” Participants rated this measure on a 10-point Likert scale ranging from 0 (*very bad*) to 10 (*very good*). Additionally, we determined potential overlap between leadership roles by asking “Is this person the same as the one you indicated earlier as task/motivational/social leader?” Based on this information, we identified whether the four leadership roles were occupied by one single leader or two, three, or four different leaders.

**Table 2:** Definitions of the four leadership roles based on the work of Fransen et al. (2014), that were presented to the participants.

Leadership role	Definition
Task leader	A task leader is in charge at work; this person helps the team to focus on goals and helps in tactical decision-making. Furthermore, the task leader gives colleagues tactical advice during work processes and adjusts them if necessary.
Motivational leader	The motivational leader is the biggest motivator at work; this person can encourage colleagues to go to any extreme; this leader also puts fresh heart into colleagues who are discouraged. In short, this leader steers all the emotions at work in the right direction in order to perform optimally as a team.
Social leader	The social leader has a leading role besides work; this person promotes good relations within the team and cares for a good team atmosphere, e.g., during breaks, in the cafeteria, or during social team activities. Furthermore, this leader helps to deal with conflicts between colleagues outside of work. This person is a good listener and is trusted by the colleagues.
External leader	The external leader is the link between our team and the people outside; this leader is the representative of our team toward the management. If communication is needed with external organizations or media, this person will take the lead. This leader will also communicate the guidelines of the management to the team.

#### Formal Leadership Quality

Immediately after rating the perceived leadership quality of a team member on a specific role, participants were asked to “Think of your formal leader and rate his/her quality on this role.” Again, this was asked for all four leadership roles with ratings ranging from 0 (*very bad*) to 10 (*very good*), which allowed for comparison between formal and peer leaders.

#### Team Identification

Participants’ identification with their team was measured with five items used by van Dick et al. (2006). This measure was rated on a 7-point Likert-scale ranging from 1 (*disagree completely*) to 7 (*agree completely*), with an example item being “I consider myself as part of my team.”

#### Team Effectiveness

Individuals’ perceived effectiveness of the team was examined with an overall scale of effectiveness by Pearce and Sims (2002) using 26 items (e.g., “The team is highly effective at implementing solutions”). Participants rated this measure on an 11-point Likert scale ranging between 0 (*disagree completely*) and 10 (*agree completely*). Here, seven subscales distinguished between output, quality, change, organizing and planning, interpersonal, value, and overall effectiveness.

#### Work Satisfaction

A total of 11 items from the Job Diagnostic Survey (van Dick et al., 2001) were used that tap into both the global work satisfaction and the satisfaction with the context. Participants rated their work satisfaction on a 7-point Likert-scale ranging from 1 (*not applicable*) to 7 (*fully applicable*). An example item is “I am generally satisfied with the kind of work I do in this job.”

#### Burnout

The extent to which the participants experienced burnout was measured using the 9-item subscale “Emotional exhaustion” of the Maslach Burnout Inventory (Maslach and Jackson, 1981) with ratings on a 7-point Likert-scale ranging from 1 (*never*) to 7 (*every day*). A sample item is “I feel emotionally drained from my job.”

### Data Analysis

Descriptive statistics (i.e., scale means and standard deviations) were computed as well as intercorrelations to test H1, H3, and H4. The proposed mediation in H2 was tested via Structural Equation Modeling (SEM) in *R*, using the maximum likelihood estimation method with robust standard errors (MLR). The degree of “fit” of the entire model was based on the following indices: the normed chi-square statistic (*χ*^2^/*df*), the comparative fit index (CFI), the Tucker-Lewis index (TLI), and the root mean square error (RMSEA). While a non-significant chi-square (*χ*^2^) implies a good fit of the data to the hypothesized model, the significance of this statistic increases with sample size. Therefore, we used the normed *χ*^2^/*df*, which indicates a good fit when its value is below 3:1 (Kline, 2005). According to Lance et al. (2006), the values of CFI and TLI ideally must be larger than 0.90 to accept a good fit, while RMSEA should be 0.08 or lower to indicate an acceptable fit.

As the impact of good leadership within the team might differ depending on whether employees are full-time vs. part-time employed, as well as upon the size of the team, we conducted regression analyses in SPSS to explore the moderating effect of type of employment and team size. Insights about these potential moderating effects can provide useful information about the applicability of shared leadership in diverse work settings.

## Results

### Descriptive Statistics

Table 1 reports the means, standard deviations, and correlation coefficients of the study variables. All correlations are significant in the predicted directions (*p* < 0.05). In the following section, the results will be reviewed as a function of the successive hypotheses.

However, before conducting all analyses for hypothesis testing, we aimed to gain insight into the extent to which leadership is currently shared within participants’ teams. More specifically, this step can offer insight into whether the four leadership roles identified by Fransen et al. (2014) are generally distributed among different team members or rather occupied by one single team member. To identify the number of peer leaders that occupied the roles of task, motivational, social, and external leader, we asked participants to indicate whether the best leader on one leadership role equaled the best leader indicated on the other leadership roles. Taken together, the results revealed that only 17.0% of the participants indicated that the four leadership roles were occupied by one single leader; 18.9% stated that these roles were taken on by two different team members; 40.9% reported that the roles were fulfilled by three different team members and 23.5% of the participants said that the four leadership roles were occupied by four different team members. In other words, an overwhelming majority of most employees (i.e., 83%) indicated that the leadership in their team was shared by different team members. Similar to sport contexts, where 70.5% of the players perceived teammates other than the team captain as more capable to fulfill these roles (Fransen et al., 2014), sharing leadership at work seems to be already acknowledged and adapted in our study sample.

### Aim 1: How Does Peer Leadership Quality Benefit the Team and Its Members?

Our first aim was to explore the benefits of PLQ for TE and team members’ work satisfaction and burnout, as perceived by each individual. In line with H1a, the correlations in Table 3 illustrates moderate positive relationships between perceived PLQ on each of the four leadership roles and the different aspects of TE (*p* < 0.01). In other words, the higher the perceived quality of task, motivational, social, and external peer leadership, the higher all seven dimensions of perceived TE. Aside from the significant contribution of each role, task leadership had the strongest relationship with TE (*r* = 0.56, *p* < 0.001).

**Table 3:** Correlations between PLQ of each leadership role and formal leadership quality.

	Peer leadership quality			
	Task leadership	Motivational leadership	Social leadership	External leadership
**Perceived leadership quality of formal leader…**				
as task leader	0.60^***^	0.41^***^	0.44^***^	0.37^***^
as motivational leader	0.43^***^	0.47^***^	0.47^***^	0.39^***^
as social leader	0.42^***^	0.44^***^	0.57^***^	0.49^***^
as external leader	0.51^***^	0.45^***^	0.45^***^	0.65^***^

*** *p* < 0.001.

Next, in line with H1b, the perceived leadership quality on all four leadership roles related positively to team members’ satisfaction with work (*p* < 0.001). Finally, in line with H1c, the results revealed significant negative correlations between PLQ and burnout (*p* < 0.05). More specifically, the better the leaders within the team, the less burnout is experienced by team members, a finding that held for each of the four leadership roles. Here, compared to all other roles, social leadership was most strongly related to burnout (*r* = −0.31, *p* < 0.001). Taken together, these findings suggest an overall positive relationship between the leadership quality within the team on all four leadership roles and TE as well as team members’ work satisfaction and burnout.

### Aim 2: Is Team Identification the Missing Link?

**Figure 2:**
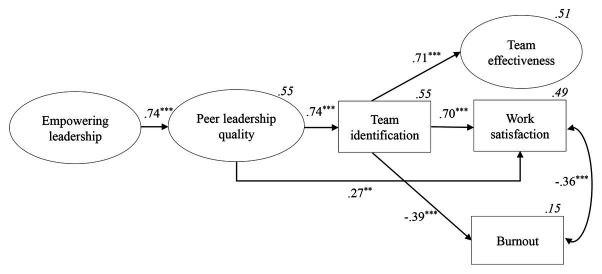
Structural model, representing the influence of empowering on peer leadership quality, with the latter in turn influencing (a) team effectiveness via full mediation of team identification, (b) burnout *via* the same full mediation of team identification, and (c) work satisfaction directly and indirectly via a partial mediation of team identification. Two covariations were included in the model: one between two subscales of team effectiveness (i.e., interpersonal and value effectiveness) and one between work satisfaction and burnout. Standardized regression coefficients are shown along each path as well as the proportions of explained variance (in italics). ^*^p < 0.05; ^**^p < 0.01; ^***^p < 0.001.

Secondly, we aimed to shed more light on the underpinning mechanisms – and, in particular the role of team identification – explaining these relationships. Table 1 reveals positive correlations between the four leadership roles and team identification (*p* < 0.001). As for mediation, the resulting model using SEM is depicted in Figure 2 and the results indicated a good model fit with *χ*^2^ = 293.32; *χ*^2^/*df* = 1.76; *df* = 166; *p* = 0.000; *TLI* = 0.93; *CFI* = 0.94; *RMSEA* = 0.08; and *SRMR* = 0.08. Based on a suggested modification index for a better model fit, we included two covariations: one between two subscales of TE (i.e., interpersonal and value effectiveness) and one between work satisfaction and burnout. Both covariations were significant (*β* = 0.62, *p* < 0.001 and *β* = −0.36, *p* < 0.001, respectively), which can be attributed to variance being explained by variables other than the ones included in the present model.^1^

First, the model revealed a significant (and strong) positive relationship between PLQ and team identification (*β* = 0.74, *p* < 0.001). Second, the model revealed significant direct relationships between team identification and all work-related outcomes, including TE (*β* = 0.71, *p* < 0.001), work satisfaction (*β* = 0.70, *p* < 0.001), and burnout (*β* = −0.39, *p* < 0.001).

The next step involved the examination of the indirect effects of PLQ to all three outcomes for the paths going through team identification. First, the results suggest a significant indirect effect from PLQ to TE (*IE* = 0.53, *p* < 0.001). This result implies a full mediation of team identification between PLQ and TE, providing support for H2a.

Second, the results suggest a significant indirect effect from PLQ to work satisfaction (*IE* = 0.52, *p* < 0.001). In contrast to the results described above, the direct path between PLQ and work satisfaction remained significant, also when team identification was added as a mediator (*β* = 0.37, *p* < 0.01). This result indicates that the relationship between PLQ and work satisfaction is only partially mediated by team identification. Therefore, H2b can only partially be confirmed.

Third, we found a significant indirect effect from PLQ to burnout (*IE* = −0.29, *p* = 0.001). This finding suggests a full mediation of team identification between PLQ and burnout, thereby confirming H2c. All standardized path coefficients and proportions of explained variance related to H2 are displayed in Figure 2.

Furthermore, regression analyses in SPSS did not reveal a moderating role of employment (part-time vs. full-time), reflected by a non-significant moderating effect of employment for TE (*F* = 26.87, *R^2^* = 0.29, *β* = 0.12, *p* = 0.34), work satisfaction (*F* = 35.14, *R^2^* = 0.34, *β* = −0.05, *p* = 0.72), and burnout (*F* = 8.76, *R^2^* = 0.12, *β* = 0.20, *p* = 0.16).

Also, team size did not have a moderating role on the impact of PLQ for TE, work satisfaction, and burnout (*F* = 22.46, *R^2^* = 0.25, *β* = −0.09, *p* = 0.24; *F* = 37.54, *R^2^* = 0.35, *β* = 0.04, *p* = 0.62; *F* = 5.55, *R^2^* = 0.07, *β* = −0.05, *p* = 0.59, respectively). We should note, though, that there was a large variety in team sizes (ranging between 2 and 280 people on one team). To ensure that our analysis for the moderating role of team size was not influenced by outliers, we also performed the analysis after eliminating 10 unusually large outliers (i.e., team sizes larger than 21). As a consequence, the results for TE and work satisfaction became significant (*F* = 17.30, *R^2^* = 0.21, *β* = −0.46, *p* < 0.01; *F* = 20.54, *R^2^* = 0.24, *β* = −0.49, *p* < 0.01), meaning that the effectiveness of PLQ was even more prominent in smaller teams. For burnout, our results remained the same and team size did not act as a moderator (*F* = 1.01, *R^2^* = 0.02, *β* = 0.12, *p* = 0.16), which implies a consistent strength of the relationship between PLQ on burnout regardless of the size of the team.

### Aim 3: The Role of the Formal Leader in Promoting Shared Leadership

With respect to H3, SEM revealed a positive relationship between EL and perceived PLQ. This finding suggests that the more the formal leader is seen as engaging in EL behaviors, the better the team members perceive the quality of leadership within the team (*β* = 0.74, *p* < 0.001). Furthermore, the moderately strong positive correlations depicted in Table 1 make clear that EL of the formal leader is related to improved PLQ on each of the four roles (*r* = 0.48, *r* = 0.47, *r* = 0.52, *r* = 0.55 for task, motivational, social, and external leadership, respectively; *p* < 0.001). In other words, the more the formal leader engages in EL, the higher the team members will rate the quality of task, motivational, social, and external peer leadership within the team, which confirms H3.

### Aim 4: The Barriers Withholding Formal Leaders From Shared Leadership

Finally, in line with H4, the correlations in Table 3 indicated significant positive and moderately strong correlations for the relation between perceived leadership quality and the formal leader’s perceived leadership quality. Notably, this finding applied to all four leadership roles (*r* = 0.37–0.65, *p* < 0.001). In other words, the higher the perceived quality of, for example, the social peer leader within the team, the more the team members perceived their formal leader as a better social leader.

## Discussion

The present study aimed to provide a deeper insight into the nature of shared leadership in organizations by investigating the leadership of team members, thereby counterbalancing the abundance of research on leadership by the formal leader (Kozlowski and Bell, 2013). More specifically, we wanted to address four different research questions to advance research in this area.

Firstly, we aimed to provide novel insights into the benefits of shared leadership. Our findings revealed significant positive relationships between the quality of peer leadership and both perceived performance (i.e., TE) and well-being indicators (i.e., work satisfaction and burnout). While these findings corroborate previous research highlighting the importance of shared leadership structures in organizations for TE (e.g., Hoch, 2007; Zhu et al., 2018), they add to the literature that the quality of the leaders within the team is also important for team members’ health and well-being. It is noteworthy that these findings held for each of the four leadership roles (i.e., task, motivational, social, and external leadership), thereby highlighting the importance of each of these roles. These results thus suggest that previous findings in sport contexts may also apply to organizations in regard to each of those outcomes (Fransen et al., 2014, 2017, 2020a).

Additionally, we tested for moderating effects of contextual variables. Until now, despite the important practical implications, most research on factors promoting or inhibiting shared leadership has neglected organizational-level or structure-based factors (Zhu et al., 2018). Our findings revealed that employment (i.e., working part-time vs. full-time) did not appear to moderate the relationship between high-quality peer leadership and all critical work outcomes. This suggests that the above findings can be generalized across diverse work settings. The link between having good peer leaders within the team and TE and well-being thus remains stable regardless of the time employees spend at work.

Next, also team size did not act as a moderator for the relationship between high-quality peer leadership and burnout. Again, this finding suggests that shared leadership consistently tempers perceived burnout regardless of the number of people constituting a team. However, this does not hold for TE and work satisfaction, where the effect of team size did appear to be stronger in smaller teams. This finding is in line with the theorizing of Zhu et al. (2018) that larger teams can mitigate the effect of shared leadership due to an increased risk of free-loading, social riding, and coordination failures. However, in a meta-analysis by Nicolaides et al. (2014) who tested the moderating role of team size in the shared leadership – performance relationship – the researchers did not find a moderating effect of team size. Resolving these contradictive findings will be particularly important as organizational teams can vary widely in size. In sum, these findings suggest a generalizable impact of shared leadership interventions on specific outcomes.

Our second aim was to shed a deeper light on the mechanisms underpinning these relationships. Our findings showed support for the SIA to leadership at various levels (Haslam et al., 2011). First, high-quality peer leadership on each of the four roles was related to higher team identification among team members. Second, the more the team members identified with their team, the higher their reported TE. Third, the more the team members identified with their team, the higher their reported work satisfaction and the lower their burnout.

The latter finding is in line with previous research on the relationship between team identification and team members’ well-being (e.g., Steffens et al., 2017). Moreover, it supports recent work on the “social cure,” highlighting the health benefits of this shared feeling of “we” and “us” (Jetten et al., 2012; Haslam et al., 2019). Yet, while most of this evidence is built on the evidence of identity leadership demonstrated by formal leaders (i.e., identity leadership; Haslam et al., 2011), the present study adds that also leaders within the team are key to cultivate a shared identity and by doing so, boost the team’s effectiveness as well as co-workers’ health and well-being. We should note, though, that the relationship between PLQ and work satisfaction appeared to be only partially mediated by team identification. PLQ thus also benefits work satisfaction in a direct way. One explanation might be that, for instance, the social leader directly influences work satisfaction by ensuring a close bond among members, providing support as a trusted person and creating a pleasant atmosphere, rather than by capitalizing on team identification. Indeed, research shows that aspects linked to what constitutes a “social leader” in this study, such as perceived collegial support, can create a favorable work atmosphere causing team members to develop positive job attitudes (e.g., Gaan, 2008; Almeida et al., 2020). For instance, a study among business managers by Bahniuk et al. (1990) revealed that job satisfaction was predicted by support from colleagues.

Our third aim was to explore the role of the formal leader in promoting shared leadership. Our findings revealed that formal leaders stimulated PLQ by engaging in EL, which in turn seems to be an asset for reaching critical work outcomes. According to a study by Kim and Beehr (2017), a possible mechanism underlying this relationship is the enhanced psychological states in team members, such as self-efficacy and psychological ownership. By encouraging an initiative among employees, such as letting them make decisions, a sense of responsibility toward their job is established, which in turn is reflected in positive workplace behavior such as peer leadership.

Fourth and finally, we took a closer look at possible barriers withholding formal leaders from implementing shared leadership. As in sport settings (Fransen et al., 2020d), we found that the higher the perceived leadership quality within the team, the more the formal leader is considered to be a good leader. Thus, empowering employees to take up leadership roles within their team has the potential to strengthen their formal leadership status instead of reducing it.

### Practical Implications of the Findings

The present study offers a more detailed understanding of the practical value of shared leadership in work teams. As a starting point, we recommend formal leaders to reconsider their management style and to empower their employees. EL, such as promoting participative goal setting or self-development, can stimulate employees to take on and fulfill peer leadership roles well. Organizations can help formal leaders in empowering their team members by providing them with specific training. First, team members need to become motivated to take up responsibility. To do this, the formal leader can formally appoint leaders within the team and give each member a participatory role which capitalizes on their own expertise. Also, demonstrating good listening skills, asking for input, and delegating authority to their employees are skills leaders can be taught in order to engage in EL (Lee et al., 2018).

Next, the findings clearly stress the positive relationship between high-quality peer leadership and both TE and well-being in teams across a wide array of organizations. These favorable outcomes further support the practical relevance of role differentiation and team identification in organizational contexts (cf. Carson, 2006).

Given the positive relationship with each of the four leadership roles, attention toward more diverse roles within teamwork is helpful, rather than simply concentrating on general or task-related leadership. With this principal guideline in mind, it is critical that team leaders identify the essential leadership roles in their organization and formally appoint the right leaders for these roles. One method by which the appropriate peer leaders can be identified is shared leadership mapping that has been proven effective in organizational teams (Fransen et al., 2015b, 2020b). In this analysis, team members rate each other’s quality on different peer leader roles, which results in clear insights about the key figures within the team. Following this, formal leaders can then invest time in the further development of those peer leaders, for example by improving their identity leadership (Haslam et al., 2011). With help of the 5R^S^ program by Fransen et al. (2020b), team members learn how to cultivate a shared social identity to grow and flourish as a team, rather than as individuals. Preliminary evidence on the impact of the 5R^S^ program in organizational teams points towards the program’s potential to improve team functioning as well as strengthening the team identity and providing individuals the opportunity to grow and flourish (Fransen et al., 2020b).

### Limitations of the Present Study

Apart from the strong points of this study, such as the inclusion of employees from a diverse set of organizations, a critical look also reveals some shortcomings. First, notwithstanding the significant and promising relationships, no causal effects can be claimed due to the cross-sectional nature of this study. Further, these relationships need to be interpreted with caution given the relatively small sample size in relation to the number of parameters in this model (*N* = 146).

Second, the theoretical framework of this study builds upon the four leadership roles derived from sports teams (Fransen et al., 2014). The findings of our study suggest that in organizations the quality of peer leaders on each of these roles is positively related to both TE and well-being, thereby providing initial confirmation on the leader categorization in sport. Nevertheless, it is likely that this four-role typology is not exhaustive. Future research is needed to identify alternate organization-specific roles for peer leaders that might even have a stronger effect on TE and well-being of employees.

Third, the study findings relied on participants’ individual perceptions about their team rather than team-level perceptions. In other words, while we are sure that the majority of the collected data stems from employees working in different teams (as they indicated different organizations), some of the participants might have worked in the same team. Therefore, the current sample did not allow us to identify clusters within our sample and to analyze our data at the team or organizational level. A fruitful avenue for future research would thus be to analyze the generalizability of our findings while controlling for team‐ or organizational-level effects.

### Future Research

Despite the increased awareness of shared leadership and its value, some unchartered areas still await future research. First, besides team size and type of employment, future research might investigate additional moderators that influence the effectiveness of shared leadership. For example, Bligh et al. (2006) argued that teams dealing with complex tasks might benefit more from shared leadership than teams dealing with simple tasks since the active inclusion of multiple members might enhance a variety of work processes.

Second, in this study participants were asked to only think of the best team member when rating PLQ. However, although other team members might not be perceived as the best leader in a specific leadership role, they can still be influential. Initial evidence from the sport context already showed that sports teams reap greater benefits of a shared leadership structure, in which more than one player fulfills a leadership role (e.g., having two task leaders instead of one; Leo et al., 2019). By mapping the entire leadership structure in the team (e.g., using social network analysis), future research can investigate whether having more leaders on each role entails higher benefits for TE and team member well-being.

### Conclusion

To conclude, this study suggests that shared leadership constitutes a promising approach to leadership for various reasons. The theoretical framework of four leadership roles derived from sports research by Fransen et al. (2014) also seems to be applicable in organizations. In fact, high-quality peer leadership in organizational teams on each of these roles appears to relate positively to work satisfaction and TE and negatively to burnout. Drawing on the SIA, these relationships were found to be mediated by team identification. Moreover, by empowering their team members to take the lead in different roles, formal leaders can stimulate high-quality peer leadership on these roles, and by doing so, are also perceived as better leaders themselves. Based on these study findings, then, it can be concluded that the perceived barriers withholding formal leaders do not necessarily hold ground and the fear of losing their own leadership status should not stop them from implementing shared leadership within their teams, even on the contrary. At the end of the day, a strong shared team identity seems to play a crucial role in successfully implementing shared leadership. This “sense of us” will be particularly important, if not necessary, to reap the benefits of teamwork within the organizations of today and tomorrow.

## Data Availability Statement

The raw data supporting the conclusions of this article will be made available by the authors, without undue reservation.

## Ethics Statement

The current study was reviewed and approved by the Social and Societal Ethics Committee at KU Leuven (G-016 09 630). The participants provided their written informed consent to participate in this study.

## Author Contributions

All authors contributed to the article and approved the submitted version. The first author CME was responsible for the data collection and writing of the first draft of this manuscript. Throughout this process, the co-authors KF and FB set up the design of the study and consistently provided feedback on the content, layout, and writing style.

### Conflict of Interest

The authors declare that the research was conducted in the absence of any commercial or financial relationships that could be construed as a potential conflict of interest.

## Footnotes

^1^Given the complexity of the model, the model fit was tested again with less parameters. More specifically, instead of testing the model with all parameters (i.e., all subscales) we included only the composite scores of empowering leadership and team effectiveness. The model fit remained acceptable with *χ^2^* = 69.65; *χ^2^/df* = 2.68; *df* = 26; *p* = 0.003; *TLI* = 0.92; *CFI* = 0.94; *RMSEA* = 0.08; and *SRMR* = 0.07.
